# Extensive Distribution of the Lyme Disease Bacterium, *Borrelia burgdorferi* Sensu Lato, in Multiple Tick Species Parasitizing Avian and Mammalian Hosts across Canada

**DOI:** 10.3390/healthcare6040131

**Published:** 2018-11-12

**Authors:** John D. Scott, Kerry L. Clark, Janet E. Foley, John F. Anderson, Bradley C. Bierman, Lance A. Durden

**Affiliations:** 1 International Lyme and Associated Diseases Society, Bethesda, MD 20827, USA; 2 Environmental Epidemiology Research Laboratory, Department of Public Health, University of North Florida, Jacksonville, FL 32224, USA; kclark@unf.edu (K.L.C.); bradleybierman@gmail.com (B.C.B.); 3 Department of Medicine and Epidemiology, School of Veterinary Medicine, University of California, Davis, CA 95616, USA; jefoley@ucdavis.edu; 4 Department of Entomology, Center for Vector Ecology and Zoonotic Diseases, The Connecticut Agricultural Experiment Station, New Haven, CT 06504, USA; john.f.anderson@po.state.ct.us; 5 Department of Biology, Georgia Southern University, Statesboro, GA 30458, USA; ldurden@georgiasouthern.edu

**Keywords:** *Borrelia burgdorferi* sensu lato, Lyme disease, ticks, birds, mammals, vertebrate hosts, ectoparasite, tick-borne pathogens

## Abstract

Lyme disease, caused by the spirochetal bacterium, *Borrelia burgdorferi* sensu lato (Bbsl), is typically transmitted by hard-bodied ticks (Acari: Ixodidae). Whenever this tick-borne zoonosis is mentioned in medical clinics and emergency rooms, it sparks a firestorm of controversy. Denial often sets in, and healthcare practitioners dismiss the fact that this pathogenic spirochetosis is present in their area. For distribution of Bbsl across Canada, we conducted a 4-year, tick–host study (2013–2016), and collected ticks from avian and mammalian hosts from Atlantic Canada to the West Coast. Overall, 1265 ticks representing 27 tick species belonging to four genera were collected. Of the 18 tick species tested, 15 species (83%) were positive for Bbsl and, of these infected ticks, 6 species bite humans. Overall, 13 of 18 tick species tested are human-biting ticks. Our data suggest that a 6-tick, enzootic maintenance cycle of Bbsl is present in southwestern B.C., and five of these tick species bite humans. Biogeographically, the groundhog tick, *Ixodes cookei*, has extended its home range from central and eastern Canada to southwestern British Columbia (B.C.). We posit that the Fox Sparrow, *Passerella iliaca*, is a reservoir-competent host for Bbsl. The Bay-breasted Warbler, *Setophaga castanea*, and the Tennessee Warbler, *Vermivora peregrina*, are new host records for the blacklegged tick, *Ixodes scapularis*. We provide the first report of a Bbsl-positive *Amblyomma longirostre* larva parasitizing a bird; this bird parasitism suggests that a Willow Flycatcher is a competent reservoir of Bbsl. Our findings show that Bbsl is present in all provinces, and that multiple tick species are implicated in the enzootic maintenance cycle of this pathogen. Ultimately, Bbsl poses a serious public health contagion Canada-wide.

## 1. Introduction

Lyme disease (Lyme borreliosis) is caused by members of the *Borrelia burgdorferi* sensu lato (Bbsl) complex [1]. This spirochetal bacterium is typically transmitted by certain blood-sucking, ixodid ticks (Acari: Ixodidae) to vertebrates, including humans. Bbsl infections present a wide spectrum of clinical manifestations from asymptomatic, to acute, to chronic, to fatal outcomes. Since Bbsl has diverse morphological forms, and is highly adaptive, this stealth pathogen can evade and slip by the immune system [2,3,4], and may instigate a persistent, life-altering spirochetosis [5,6]. Acarologists have documented 40 tick species parasitizing vertebrate hosts in Canada [7,8,9]; however, many of these tick species have not been tested for the presence of Bbsl.

Ticks have been present in Canada since antiquity. Based on phenology, migratory birds have been transporting ticks into Canada since the last ice age 10,000 years ago. Each year, neotropical and southern temperate songbirds transport ticks across the Canada-U.S. border into Canada during northward spring migration, and widely disperse bird-feeding ticks country-wide [10,11,12,13,14,15]. Likewise, long-distance migrating passerines (Passeriformes) import *Amblyomma* ticks, such as *A. americanum* (lone star tick), *A. dissimile*, *A. humerale*, *A. maculatum* (Gulf Coast tick), *A. longirostre*, and *A. rotundatum* into Canada from as far south as the northern part of South America [9,10,11,16,17]. The home ranges of *A. dissimile* and *A. longirostre* in north-central South America coincide with the migration patterns and wintering grounds of certain neotropical songbirds and, with plentiful energy reserves and southerly tailwinds, these passerine migrants provide rapid transport for *Amblyomma* ticks to Canada.

Using a mathematical computer model, researchers recently purported that *A. americanum* has the potential to establish in Montreal, Canada [18]. In contrast, our 3-year overwinter survival study at Fergus, Ontario, reveals that *A. americanum* larvae do not overwinter in southwestern Ontario (J.D.S., unpublished data). Even though *A. americanum* nymphs and adults may overwinter, larvae do not. When migrating songbirds transport larval and nymphal *A. americanum* ticks into Canada, they release fully engorged larvae and nymphs into the leaf litter and humus layer. During the late spring and early summer, replete larvae and nymphs undergo ecdysis (molt) to become nymphs or adults, respectively, and parasitize domestic and wildlife animals [19]. During the blood meal, this invasive tick species commonly transmits foreign pathogens to vertebrates, including humans.

Certain areas across Canada have Lyme disease foci where multiple species of ticks are present. For instance, Scott et al. documented 9 tick species in the vicinity of Kenora, Ontario, and eight species tested positive for Bbsl [20]. From a medical standpoint, five of these tick species are known to bite humans [21].

The primary aims of this study were to (i) ascertain the distribution of Bbsl-positive ticks across Canada; (ii) establish the prevalence of Bbsl in ticks parasitizing avian and mammalian hosts in certain regions; (iii) determine whether any tick species shifted their home range; and (iv) assess the number of tick species that potentially bite humans and inflict Lyme disease.

## 2. Materials and Methods

### 2.1. Tick Collection

Ticks were obtained from hosts by various means. They were collected from birds and mammals by bird banders, wildlife rehabilitators, and biologists. Ticks were also obtained from citizens who removed them from themselves, their pets, birds, and animals killed on roads. When ticks were collected by bird banders, birds were promptly aged, weighed, sexed, and then released to the wild. Attached ticks were removed with fine-pointed, stainless steel forceps. If ticks were damaged upon removal, they were placed in 2 mL micro tubes (Sarstedt) containing 94% ethyl alcohol. Otherwise, live ticks were put in a transparent, round-bottom, 8.5 mL polypropylene tube (15.7 mm × 75 mm, round based) (Sarstedt). The mouth of the tube was covered with tulle netting (3 cm diameter) to allow ventilation for ticks. A polyethylene push cap, with a 7-mm hole, was placed into the mouth of the tube to secure the tulle netting, and prevent tick escapes. Each tube, which contained the ticks from one host, was placed in a double-zipped plastic bag with a slightly moistened paper towel to maintain high humidity. All ticks were sent to the lab for identification (J.D.S.). With the exception of two specimens, these ticks were identified using taxonomic keys for larvae [22], nymphs [23], and adults [24]. Since all *Amblyomma* larvae molted to nymphs, nymphal keys were used for this genus [25,26,27]. Any ticks that were fully engorged were held to molt to the next life stage or, in the case of females, to lay eggs. *Ixodes* species were exposed to a long-day photoperiod of 16:8 h (L:D). Bird-feeding *Amblyomma* ticks from the Neotropics were held at a photoperiod of 12:12 h (L:D). Complete records (i.e., geographic location, tick collection date, tick species, developmental life stage, and host species) were logged for each tick collection.

We tabulated the life span of one generation of the groundhog tick, *Ixodes cookei*. We held eggs, larvae, nymphs, males, and females in outdoor housing units at 85% to 100% relative humidity year-round to see how long each life stage would live.

Ethical approval and informed consent are not required because removal of ticks is not an invasive procedure, and no personal identifiable data were obtained.

### 2.2. Spirochete Detection

After identification, ticks were sent to three different laboratories for Bbsl testing because the first two institutions had policy changes during this study, and halted tick testing. The 3 sequential phases were: February 2013–June 2013 (J.F.A.), July 2014–June 2015 (K.L.C.), and July 2015–November 2016 (J.E.F.). During the first phase (J.F.A.), bacteria from live ticks were cultured in Barbour-Stoenner-Kelly (BSK) medium, whereas dead ticks were processed directly for DNA extraction followed by PCR (polymerase chain reaction) amplification. The DNA detection protocol was previously described [28,29,30]. Even though Persing et al. employed both the flagellin (*flaB*) gene and the major outer surface protein A (*OspA*) gene [28], this lab (J.F.A.) only used the *OspA* gene. Bona fide negative and positive controls were utilized.

In the second phase of our study, ticks were put directly in 94% ethyl alcohol, and forwarded to the lab (K.L.C.). These ticks were tested using a nested PCR that amplifies a portion of the flagellin (*flaB*) gene of Bbsl, with slight variations from a previously described protocol [31]. The primary PCR assay, which targets a 497-nt fragment of the *flaB* gene, used the following primers, 271F: 5′-AAG-GAA-TTG-GCA-GTT-CAA-TCA-GG-3′ and 767R: 5′-GCA-TTT-TCT-ATT-TTA-GCA-AGT-GAT-G-3′. The secondary (nested) PCR amplified a 437-nt internal fragment using primers 301F: 5′-ACA-TAT-TCA-GAT-GCA-GAC-AGA-GG-3′ and 737R: 5′-GCA-TCA-ACT-GTA-GTT-GTA-ACA-TTA-ACA-GG-3′.

In the third phase, ticks were directly put in 94% ethyl alcohol, and sent promptly by courier to the lab (J.E.F.). The protocol used for detecting Bbsl is outlined in Barbour et al. [32].

Since the occurrence of *B. miyamotoi*, a relapsing fever group spirochete, is deemed to be rare (<1%), we did not screen ticks for this microorganism. The PCR primers that we used were designed and applied in the study to specifically detect known species in the *B. burgdorferi* sensu lato complex.

In this study, the infection rate is the number of ticks infected with Bbsl divided by the number of ticks tested.

### 2.3. Molecular Tick Identification of Ixodes cookei

In order to confirm the identification of *I. cookei* collected in southwestern British Columbia, we used molecular methodology, and barcoded a larval tick (17-5A85L) hatched from the eggs of a gravid female, which was collected at North Saanich, Vancouver Island. The molecular identification was conducted at the Centre of Biodiversity Genomics (CBG), University of Guelph with accession number BIO-18-060. The DNA extract is being held at −80 °C at the same location. The collection data and barcode sequence is stored in BOLD, and can be accessed in the BOLD dataset at: dx.doi.org./10.5883/DS-IGAK.

## 3. Results

### 3.1. Tick Collection

During the 4-year study period (2013–2016), a total of 1265 ixodid ticks representing 27 tick species belonging to four genera (*Amblyomma*, five; *Dermacentor*, three; *Haemaphysalis*, one; *Ixodes*, 18) were collected from avian and mammalian hosts Canada-wide (Table 1, Table 2, Table 3, Table 4, Table 5 and Table 6). The tick–host associations consist of (1) 16 tick species taking a blood meal from birds; (2) 25 tick species feeding on mammals; and (3) 10 tick species that parasitize both birds and mammals. Overall, 14 of 27 tick species are known to bite humans (Table 1) [21]. However, *Ixodes brunneus* is a true bird-associated tick [23], and parasitism of humans by this ectoparasite would be very unlikely.

**Figure 1:**
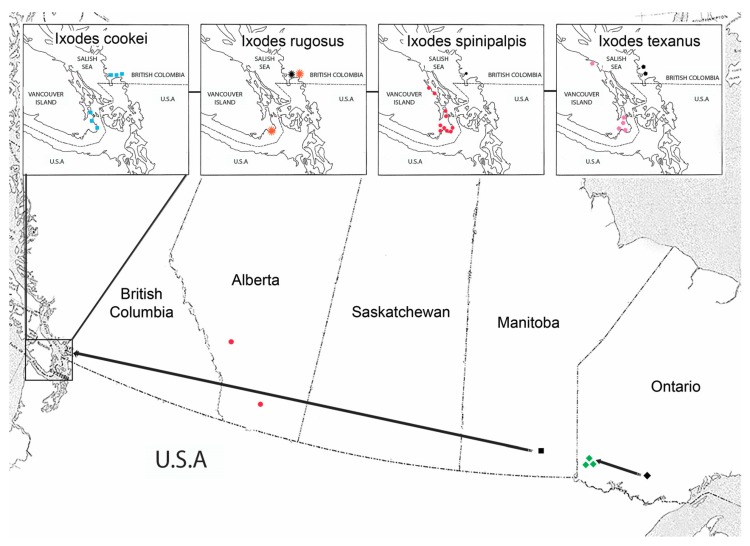
Map of western Canada showing baseline and current biographical distribution of five *Ixodes* spp. Black symbols represent benchmark sightings for the tick species [7,8]. Color symbols represent current sighting for *I. cookei* (blue square), *I. gregsoni* (green diamond), *I. rugosus* (orange star), *I. spinipalpis* (red dot), and *I. texanus* (mauve polygon).

Eight co-infestations (seven double, one triple) were identified (Table 6). The triple co-infestation consisted of *Ixodes angustus* (male, female), *Ixodes pacificus* (western blacklegged tick) (nymph, Bbsl-positive), and *Ixodes spinipalpis* (nymph). When we looked at earlier tick–host studies [7,33], and compared their findings with our dataset, we discovered a number of range extensions for certain tick species, namely *I. cookei*, *Ixodes gregsoni* (a mustelid-feeding tick), *I. spinipalpis*, *Ixodes rugosus*, and *Ixodes texanus* (raccoon tick) (Figure 1). From a medical standpoint, we collected an *I. spinipalpis* nymph from a human; the nymph tested negative for Bbsl. Because *I. spinipalpis* has vector competence for Bbsl, this tick species has the potential to transmit Lyme disease spirochetes to people.

In this study, we report many novel host records for ticks on birds and mammals (Table 6). The two focal study areas comprise: (a) Pacific region: Metchosin-Victoria-Vancouver-Maple Ridge in southwestern British Columbia (Table 2 and Table 3) and (b) Eastern region: London-St. Thomas-Simcoe-Toronto in southern Ontario (Table 4 and Table 5). In southwestern B.C., 22 *I. cookei* were collected from three different mammal species (American mink, Pacific raccoon, and striped skunk). In addition, *I. pacificus*, *Ixodes rugosus*, *Ixodes soricis* (shrew tick), *I. spinipalpis*, and *I. texanus* were collected from mammals on Vancouver Island (Figure 1). Excluding *I. soricis* and including the avian coastal tick, *Ixodes auritulus*, we put forward a 6-tick, enzootic maintenance transmission cycle of Lyme disease spirochetes in southeastern region of Vancouver Island.

In southwestern Ontario, 27 larval and nymphal specimens of *A. longirostre*, which are native to the Neotropics, were collected from neotropical songbirds during northward spring migration.

In order to answer a long-standing question of how long *I. cookei* live, we determined the longevity of one generation of *I. cookei*: 4201 days (11.51 years). The breakdown for the developmental life stages (egg, larva, nymph, and adult) was 55 days, 614 days, 919 days, and 2613 days (female), respectively. This dataset provides rudimentary information on the sustainment of *I. cookei*, and represent the longest living individuals for each developmental life stage.

We provide two novel tick–host records for blacklegged ticks, *Ixodes scapularis* (northern populations previously considered *Ixodes dammini*), on passerine birds. A fully engorged *I. scapularis* nymph was collected from a Bay-breasted Warbler, *Setophaga castanea*, on 15 May 2015 at Ste-Anne-de-Bellevue, Québec during spring migration. This replete nymph underwent ecdysis to a female in 72 days. Likewise, two *I. scapularis* larvae were collected from a hatch-year Tennessee Warbler, *Vermivora peregrina*, at Ruthven Park, Ontario on 1 September 2013 during fall migration.

**Figure 2:**
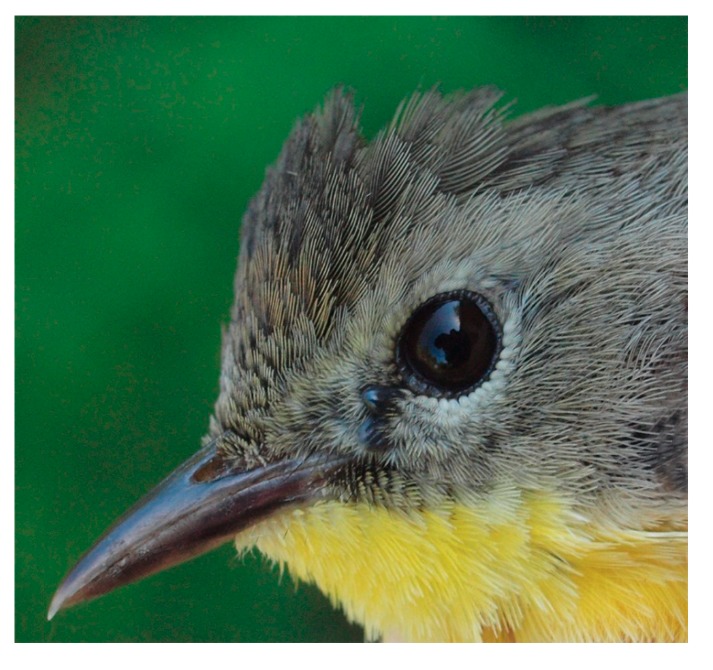
Common Yellowthroat parasitized by two engorging *I. scapularis* nymphs, both together, anterior to left eye. One of the two nymphs was positive for *B. burgdorferi* sensu lato. Photo credit: Simon Duval.

In the Pacific region, the most common avian host parasitized by ticks was the Song Sparrow, whereas in the Eastern region, the Common Yellowthroat, a ground-foraging passerine, was most frequently parasitized by ixodid ectoparasites (Figure 2).

**Figure 3:**
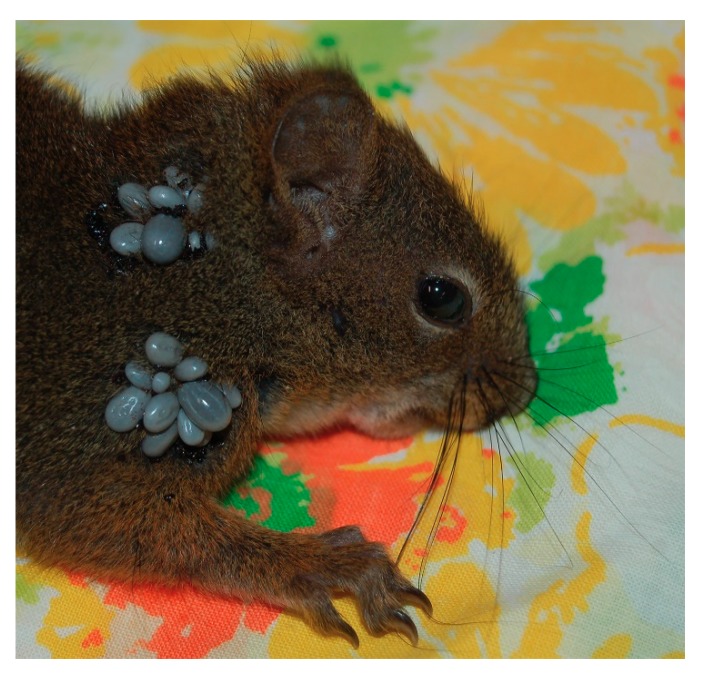
Red squirrel parasitized by 29 *I. angustus* females. Five males were copulating on the ventral surface of five females, and are not visible. Some of the *I. angustus* ticks on this host were positive for *B. burgdorferi* sensu lato. Photo credit: Christina Carrieres.

We report 34 *I. angustus* ticks (29 females, five males) parasitizing a red squirrel at East Sooke, British Columbia on 28 October 2013; this heavy tick infestation constitutes the first documentation of Bbsl-positive *I. angustus* on this sciurid species in Canada (Figure 3).

We collected 24 *I. rugosus* nymphs from a river otter at Esquimalt, B.C.; not only is this collection a new host record, it is the first account of this tick species on Vancouver Island. Additionally, we collected a single *I. texanus* female from a Pacific raccoon at Colwood, Vancouver Island; this collection is the southernmost record in British Columbia.

### 3.2. Molecular Tick Identification of Ixodes cookei

A gravid *I. cookei* female was collected from a striped skunk on 15 June 2017 at Saanich, Vancouver Island, B.C. This female laid eggs that hatched into larvae (BIO-18-060), and one of these larvae was barcoded at BIO (Biodiversity Institute of Ontario), University of Guelph, Ontario. The barcoding analysis confirmed the identification as *I. cookei*, and the nucleotide sequence was deposited in GenBank with accession number: MH338173.

Based on a sequence length of 620 bp, this larva had a 99.8% similarity match to three other *I. cookei* in BOLD. This molecular tick identification verifies the establishment of *I. cookei* in southwestern B.C.

### 3.3. Spirochete Detection

We sampled a wide cross section of ticks collected coast to coast, and tested 18 tick species for Bbsl (Table 1). Of the 18 tick species tested, 15 species (83%) were positive for Bbsl and, of these systemically infected ticks, 6 species bite humans. In the Western region, we collected 25 *I. rugosus* nymphs (one from a striped skunk, and 24 from a river otter), but none was positive for Lyme disease spirochetes. It is noteworthy that 11 (35%) of 31 *I. angustus* adults were positive for Bbsl. In the Pacific coastal region, we detected Bbsl in three tick species parasitizing birds (Table 2) and, likewise, in seven tick species infesting mammals (Table 3).

In southwestern B.C., 5 (23%) of 22 *I. cookei*, which were collected from three different mammalian hosts (i.e., Pacific raccoon, American mink, and striped skunk) were positive for Bbsl (Table 3). Specifically, the biogeographic breakdown for Bbsl-positive *I. cookei* in southwestern B.C. was Mainland, 2 and Vancouver Is., 3. Our data provide newfound evidence of Lyme disease spirochetes in *I. cookei* in far-western Canada.

A single *I. soricis* tick was collected from a roadkill vagrant shrew on the southern shoreline of Vancouver Island, B.C.; it was not tested for Lyme disease spirochetes, but instead, kept as a voucher specimen.

Notably, 17 (77%) of 22 *I. auritulus* larvae collected from a Fox Sparrow, *Passerella iliaca*, were positive for Bbsl, which suggests that this ground-frequenting songbird is a reservoir-competent host.

In the eastern Canadian region, we detected Bbsl in two tick species parasitizing birds (Table 4) and, similarly, in four species infesting mammals (Table 5). Notably, 16 (59%) of 27 *I. scapularis* nymphs collected from ground-foraging songbirds during northward spring migration were infected with Lyme disease spirochetes. Additionally, we collected Bbsl-infected *I. scapularis* larvae from a northern short-tailed shrew, which reaffirms that this small mammal is a reservoir-competent host.

In the laboratory (J.F.A.), the majority of cultures became contaminated, and only one culture produced motile spirochetes. This culture was PCR positive for Bbsl, but it was not sent for DNA sequencing.

Of epidemiological significance, 1 of 27 *A. longirostre* ticks was positive for Bbsl; this is the first report of a Bbsl-positive *A. longirostre* parasitizing a bird in North America. In addition, we provide the first report of Bbsl in *I. brunneus* and *I. texanus* in Canada.

## 4. Discussion

We detected Bbsl in 15 of 18 tick species tested, and unveiled multiple host records nationwide (Table 6). In certain regions of Canada, we encountered several tick species in a complex enzootic maintenance cycle of Bbsl. In one particular locality on the Pacific coast, we encountered several tick species parasitizing avian and mammalian hosts, and some of these host-seeking ticks bite humans. Surprisingly, we observed instances where ticks had shifted their host range, or were noted for the first time. Because we collaborated closely with biologists and wildlife rehabilitators in certain locations, we were able to collect a proportionally larger number of ticks in these areas. From the Atlantic Ocean to the Pacific Ocean, we detected the Lyme disease spirochete in a continuum of 15 different tick species.

### 4.1. Tick Vector Competency for B. burgdorferi Sensu Lato

In total, eight tick species in our study are known to be competent vectors of Lyme disease spirochetes. They include *Ixodes affinis*, *I. angustus*, *Ixodes dentatus* (rabbit-associated tick), *Ixodes minor*, *Ixodes muris* (mouse tick), *I. pacificus*, *I. scapularis*, and *I. spinipalpis* [38]. Because all motile life stages (larvae, nymphs, and adults) of *I. auritulus* had significant Bbsl infection prevalence, and this tick species is exclusively parasitizes birds, we further indicate that *I. auritulus* has vector competence for Bbsl (Table 2) [39].

### 4.2. Ticks as Bridge Vector for B. burgdorferi Sensu Lato

In this study, we uncovered eight tick co-infestations (seven double species, one triple species) on vertebrates. When a Bbsl-infected tick parasitizes its host, it can transmit spirochetes during its blood meal. If the host is spirochetemic, it can then act as a source of Lyme disease spirochetes to other cofeeding ticks, either the same tick species or a different tick species. When these engorging ticks have fed to repletion, they drop off and undergo ecdysis before they can transmit spirochetes to suitable vertebrate hosts, including humans. Depending on the developmental life stage of the tick, the molt normally takes five to eight weeks. Both *I. pacificus* and *I. scapularis* are noted as the primary bridge vectors of Lyme disease spirochetes to humans [40].

### 4.3. Dispersal of Vector-Borne Pathogens

During this study, four genera (i.e., *Amblyomma*, *Dermacentor*, *Haemaphysalis*, and *Ixodes*) of ticks were collected from wild birds and terrestrial mammals across Canada. With the exception of *I. affinis*, *I. minor*, and *Amblyomma* species, the remainder of tick species are established and survive successfully in Canada. Migratory songbirds import bird-feeding ticks into Canada annually during spring migration, and these songbird-transported ticks and associated avifauna may be infected with a wide range of vector-borne, zoonotic pathogens. These bird parasitisms include Bbsl [10,11,12,13,14,15,41], *Anaplasma phagocytophilum* [12,42], *Babesia* spp. [42], *Bartonella* spp. [43], and vector-borne viruses [44,45]. Neotropical passerines migrate across national and intercontinental borders, and become long-range vectors for any zoonotic pathogen that they harbor. Overall, dispersal of Bbsl-infected ticks along migration routes is an important mechanism in the establishment of new endemic foci of tick-borne diseases [46].

### 4.4. Metchosin-Victoria-Vancouver-Maple Ridge Region

In southwestern B.C., Lyme disease spirochetes were detected in a wide range of tick species and associated vertebrate hosts that serve as reservoirs (Figure 1 and Table 2 and Table 3). In this coastal region, we detected Bbsl in six different tick species, namely *I. angustus*, *I. auritulus*, *I. cookei*, *I. pacificus*, *I. spinipalpis*, and *I. texanus* (Table 6). Ecologically, three tick species (i.e., *I. auritulus*, *I. pacificus*, and *I. spinipalpis*) feed on wild birds, whereas five tick species (i.e., *I. angustus*, *I. cookei*, *I. pacificus*, *I. spinipalpis*, and *I. texanus*) parasitize mammals. In order to form an interconnecting link between birds and mammals, *I. pacificus* and *I. spinipalpis* provide a bridge for Lyme disease spirochetes; this bridge also extends to humans. Collectively, five of these tick species bite humans, specifically *I. angustus* [47,48], *I. cookei* [48,49,50], *I. pacificus* [51], *I. spinipalpis* [52,53,54,55], and *I. texanus* [48,49]. Any of these tick species have the potential to be a vector of Lyme disease spirochetes to humans in far-western Canada.

Since passerines and raptors are parasitized by bird-feeding ticks, they provide an interconnecting link for Bbsl back and forth across the Salish Sea. More epidemiologic details on avian and mammalian hosts in this region are provided in Table 6. In the Pacific Northwest, *I. auritulus* is the most frequently occurring tick species parasitizing wild birds, namely raptors [30] and passerines [39]. In addition, several mammals act as reservoirs in an ongoing enzootic maintenance cycle of Bbsl along B.C.’s Pacific coast (Table 3).

Along the Pacific B.C. coast, *I. auritulus* plays a vital role in maintaining the presence of Lyme disease spirochetes. In all, 49 (48%) of 102 *I. auritulus* (larvae, nymphs, and adults) in this study were infected with Bbsl. Similarly, Scott et al. detected Bbsl in 31% of *I. auritulus* [39]. Although *I. auritulus* only parasitizes avifauna, both birds and mammals eat these ixodid ectoparasites, and may become systematically infected by oral inoculation [56].

### 4.5. London-St. Thomas-Simcoe-Toronto Region

The most commonly occurring tick species in our study was the blacklegged tick (Table 4 and Table 5). East of the Rocky Mountains, *I. scapularis* [57,58] is the most prevalent tick species on passerine migrants during northward spring migration (Table 4). Ecologically, the peak questing activity of nymphs coincides with peak spring migration of northbound passerine migrants.

Bbsl-positive *I. scapularis* larvae were collected from a northern short-tailed shrew, which reinforces that this insectivore is a reservoir host. This parasitism is consistent with other researchers who found that this shrew is a reservoir-competent host [59,60,61]. Because there is no transovarial transmission of Bbsl in *I. scapularis* females [62], we are confident that the source of infection was this spirochetemic host. This high-energy insectivore burrows through leaf litter and the humus layer, and has ample opportunity to become parasitized by Bbsl-infected *I. scapularis* immatures, especially in a Lyme disease endemic area.

### 4.6. Geographical Distribution of Ticks

We document new Canadian foci where certain tick species have been collected for the first time. Our novel tick findings may have been oversights by earlier tick studies [7,8] or unnoticed biogeographical shifts. Any ticks that are not transported by wild birds must have a terrestrial mode of transportation to occupy a new area.

East of the Rocky Mountains, bird-feeding *Ixodes* ticks, such as *I. affinis* [14,15,63], *I. brunneus* [14,15], *I. dentatus* [14,15], *I. minor* [36], and *I. scapularis* [11,13,14,15] can be transported long distances by migrating songbirds. Along the Pacific coast, *I. auritulus*, which is exclusively on birds [23], can be transported from the lower mainland to offshore islands, and established in coastal ecosystems. Realistically, any bird-feeding ticks (e.g., *I. auritulus*, *I. spinipalpis*, and *I. pacificus*) can be transported by wild birds from British Columbia’s mainland to Vancouver Island, the Gulf Islands, and insular tracts of land.

In eastern Canada, we noticed a biogeographical shift in *I. gregsoni.* An archetype study reported this mustelid tick at Ignace, Ontario [33]. We report *I. gregsoni* 200 km further west at Jim Lake, Ontario (Figure 1).

### 4.7. Mammal Parasitisms Reflect Established Ticks

This study provides many mammalian, tick–host associations across Canada. The highlights of these first-time records of mammal parasitisms are reported in Table 6. Since terrestrial mammals have a localized home range, they typically signify that a given tick species is established in the vicinity.

In the present study, 25 (93%) of the 27 tick species parasitize mammalian hosts, including humans. Not only do mammal parasitisms signify the presence of a tick species in a locality, Bbsl-positive ticks indicate that Lyme disease spirochetes are cycling enzootically in the area. These established populations may be hundreds of kilometres from where the zoonotic pathogens were acquired. In particular, one established population of blacklegged ticks on Corkscrew Island, which is located in northwestern Ontario, has a Bbsl infection prevalence of 73%; this insular location has the highest mean infection prevalence of Lyme disease spirochetes in *I. scapularis* adults ever reported in Canada [64].

In the present tick-host-pathogen study, all of the *I. dentatus* and *Ixodes marxi* (eastern squirrel tick) were negative for Bbsl (Table 1). However, other studies report Bbsl-positive *I. dentatus* and Bbsl-positive *I. marxi* [14,65].

### 4.8. Ixodes cookei Established on Vancouver Island

We provide the first report of *I. cookei* in far-western Canada and the West Coast of North America. We collected five *I. cookei* (one female, one nymph, three larvae) from a Pacific raccoon at North Saanich; this tick collection is the first record of *I. cookei* west of Manitoba (Table 6). Additionally, a gravid female was collected from a striped skunk on Vancouver Island, and it laid eggs that subsequently produced viable larvae. One of these larvae was barcoded for tick identification, and confirmed as *I. cookei*. The parasitism of a terrestrial mammal is the only viable mode of passage to Vancouver Island. Since *I. cookei* is not a bird-feeding tick [23], it must have been introduced via a terrestrial host that serves as a reservoir. Not only were Bbsl-positive *I. cookei* collected on Vancouver Island, they were collected from mammals in the lower Fraser Valley on the B.C. mainland. In the case of *I. cookei* on Vancouver Island, we hypothesize that a traveller from an indigenous area in central or eastern Canada took a terrestrial animal, such as a dog, to Vancouver Island, B.C., and the mammalian host was infested with a gravid *I. cookei* female and a male. During the trip westward, the female mated with the male and, upon arrival, the replete female dropped off into the leaf litter in a woodland habitat and, subsequently, laid eggs that hatched into viable larvae. Thus, a new *I. cookei* population was initiated on Vancouver Island. Since *I. cookei* had not previously been discovered in southwestern British Columbia, we contend that this previously undetected population became established recently, and is clearly not attributed to climate change [66].

### 4.9. Composite 6-Tick Enzootic Cycle of Bbsl on Vancouver Island

**Figure 4:**
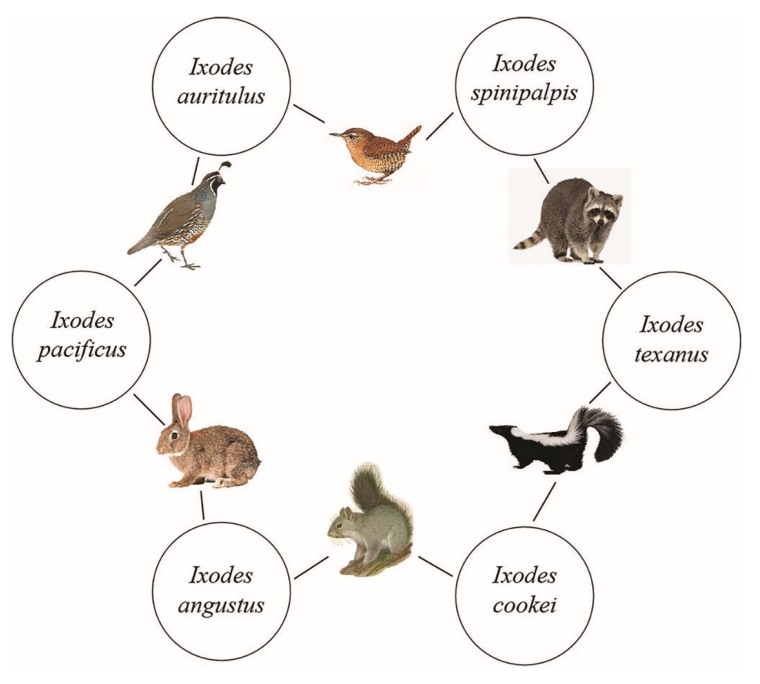
A 6-tick cycle of *Borrelia burgdorferi* sensu lato on Vancouver Island, B.C. Collectively, these six tick species form an interconnecting link between multiple hosts in a multipart enzootic maintenance cycle of Bbsl. Five of these tick species bite humans.

Whenever there are two or more tick species feeding concurrently on a host, they can transmit Bbsl, via the reservoir host, from one cofeeding tick species to another cofeeding tick species. Alternatively, one tick species can infect a reservoir-competent host and, after the blood meal, another tick species can subsequently acquire Bbsl from this spirochetemic host. Regardless of the sequel of feeding, co-infestations provide a direct way to maintain an enzootic transmission cycle of Lyme disease spirochetes. Using six vertebrate hosts and six tick species (i.e., *I. angustus*, *I. auritulus*, *I. cookei*, *I. pacificus*, *I. spinipalpis*, and *I. texanus*) from this study, we show a complete enzootic circuit of Bbsl from one tick species to another (Figure 4). With the exception of *I. auritulus*, these tick species bite people. In essence, this continual interconnecting link fulfills a multifaceted enzootic maintenance cycle of Bbsl.

### 4.10. Amblyomma Ticks Transported to Canada

*Amblyomma* ticks are transported into Canada by neotropical songbirds from the Neotropics. In order for these ixodid ectoparasites to sustain the transcontinental flight, they must remain attached for sustained periods of time. In the case of *A. americanum*, larvae typically feed 3 to 6 days, whereas nymphs commonly feed 4 to 7 days (J.D.S., unpublished data). Because many neotropical songbirds are marathon flyers, they are capable of transporting neotropical ticks long distances. During the flight, bird-feeding ticks gradually take a blood meal, and are fully engorged when they arrive in Canada. Based on these circumstantial factors, certain neotropical songbirds (e.g., flycatchers, thrushes, and warblers) transport slow-feeding *Amblyomma* ticks thousands of kilometres to Canada.

With respect to *Amblyomma* ticks, we collected *A. americanum*, *A. dissimile*, *A. longirostre*, *A. maculatum*, and *A. rotundatum* from neotropical and southern temperate songbirds in central and eastern Canada during northward spring migration. Additionally, Scott et al. previously reported an *A. americanum* (nymph) on a neotropical songbird (Swainson’s Thrush) in northwestern Alberta during spring migration [13]. Based on winter hardiness studies in southwestern Ontario, which is the southernmost region in Canada, we determined that *A. americanum* larvae do not overwinter (J.D.S., unpublished data). After migration, songbird-transported *A. americanum* larvae and nymphs will undergo ecdysis in late spring, and subsequently bite vertebrates, including people. However, the larvae will neither survive subzero Canadian winters, nor sustain an *A. americanum* population. For example, a neotropical songbird can import an *A. americanum* nymph into Canada in May during northward spring migration. Over the next 35 to 60 days, this replete tick will molt to either a male or a female, and starts host-seeking. A female will typically bite dogs, outdoor cats, and humans in August. Additionally, songbirds can introduce ticks infected with pathogens from southern climes that cause invasive zoonoses in domestic and wildlife mammals in Canada. Based on the geographic origin of *Amblyomma* spp., it is highly unlikely that these invasive ticks from tropical or semi-tropical regions will colonize in Canada.

### 4.11. Long-Distance Transport of Neotropical Ticks to Canada

Notably, a Bbsl-positive *A. longirostre* larva was collected from a Willow Flycatcher at Toronto, Ontario; this bird parasitism provides the first report of Lyme disease spirochetes in this neotropical tick species in North America. The immature stages preferentially parasitize passerine birds, while adults are found on rodents. As adults, *A. longirostre* have the potential to transmit pathogens to other large mammals, including humans.

Overall, we collected 27 *A. longirostre* larvae and nymphs from neotropical songbirds during spring migration. A combination of ecological factors facilitate the fast, long-distance movement of *Amblyomma* ticks from Brazil to Canada. From a zoogeographical perspective, *A. longirostre* ticks have a home range in north-central South America; Brazil is a significant part of this indigenous area [26]. As well, many neotropical passerines, which migrate to the northern boreal forest, have their wintering grounds in Brazil. Certain songbirds, such as the Willow Flycatcher fly long distances during migratory flight. In fact, they will fly up to 8000 km to Canada during northward spring migration [67]. During fall migration, a Willow Flycatcher flew 2217 km for 48 h nonstop from Harrison, Illinois to Minatitlan, Veracruz, Mexico with a flight pace of 45 km/h, or 1109 km/day [68]. At this flight pace, a Willow Flycatcher could fly from Brazil to Canada, a distance of 5240 km, in 4.7 days. With adequate food reserves and sustained southerly tailwinds, a Willow Flycatcher has the potential to transport *Amblyomma* ticks from Brazil to Canada.

### 4.12. Avian Host Records for I. scapularis

We document the first host records of *I. scapularis* parasitizing a Tennesse Warbler and a Bay-breasted Warbler. Both of these neotropical songbirds have their breeding range primarily in the northern boreal forest. With respect to the Bay-breasted Warbler, a fully engorged *I. scapularis* nymph was collected from this ground-foraging songbird at Ste-Anne-de-Bellevue, Québec during north-bound spring migration; this parasitism is a new tick–host record.

During fall migration, two *I. scapularis* larvae were collected from a hatch-year Tennessee Warbler. This bird parasitism indicates that this fall migrant was parasitized by the two larvae at a more northern location. Since one larva was Bbsl-positive, the spirochetal infection could have been transmitted transovarially via the mother bird to her eggs, and passed onward to her offspring and, thus, to this fledgling. When the fledgling was parasitized by two *I. scapularis* larvae, one larva became infected. Since the Bbsl-infected larva had not had a previous blood meal, the mother bird could be a competent reservoir. Although it is possible that maternal–neonatal transmission of Lyme disease spirochetes occurred between the mother bird and its offspring, it is more likely that the fledgling was bitten by a Bbsl-infected tick while foraging for food after leaving the nest, and became spirochetemic. In essence, immature stages of *I. scapularis* parasitize migratory songbirds during bidirectional migrations. Nymphs typically parasitize passerine migrants in the late spring, whereas *I. scapularis* larvae commonly parasitize songbirds in late summer and early fall.

### 4.13. Novel Bird Parasitisms

Ticks normally bite wild birds on the head and neck [11]; however, we document a tick parasitizing its avian host in the buccal cavity. Specifically, a fully engorged *I. scapularis* nymph was detached from the base of the mouth of an American Kestrel nestling [37]. This 3-week-old nestling was collected at Mirabel, Québec shortly after leaving the nest. This bird parasitism reveals the first collection of *I. scapularis* from an American Kestrel in Canada.

Migratory songbirds, which are heavily infested with ticks, have the potential to initiate new foci of ticks hundreds of kilometers from their original source [42,55,69]. Even though spring passerine migrants transport ticks to northern latitudes, these engorged ticks may not molt to the next live stage. Each tick species has its own photoperiod requirements to activate and undergo ecdysis [19]. In the case of *I. scapularis*, this tick species requires at least 14 hours of daylight for larvae and nymphs to molt to the next developmental life stage [66]. In actuality, the expansion and establishment of *I. scapularis* in northern areas is limited by photoperiod.

In this study, we detected Lyme disease spirochetes in 17 (77%) of 22 *I. auritulus* larvae feeding on a Fox Sparrow. The presence of Bbsl in a replete *I. auritulus* larva does not automatically confirm that a bird is a reservoir-competent host. However, since *I. auritulus* larvae attached to Fox Sparrows have been consistently positive for Bbsl, it is highly likely that the Fox Sparrow is a competent reservoir of Lyme disease spirochetes. More specifically, our results are congruent with other studies that show Bbsl-positive *I. auritulus* larvae collected from Fox Sparrows [11,35]. In order to confirm reservoir competency in birds, Richter et al. conducted a xenodiagnostic study using spirochete-free *I. scapularis* larvae to show that certain passerines, such as the American Robin, are reservoir-competent hosts of Bbsl [70]. Since transovarial transmission of Bbsl is not apparent in *I. auritulus* females, we postulate that Fox Sparrows are reservoir-competent hosts.

*Ixodes auritulus* harbors a diversity of Bbsl genomospecies. For example, Scott et al. documented *B. burgdorferi* sensu stricto, plus three other genotypes, in bird-feeding *I. auritulus* ticks collected in southwestern B.C. [13]. In the same province, Scott et al. identified *Borrelia lanei* (formerly *Borrelia* genomospecies 2), which is another member of the Bbsl complex, in *I. spinipalpis* adults collected from an eastern cottontail along the southern fringe of Vancouver Island (Table 6) [34]. Additionally, Scott & Foley discovered *Borrelia americana*, a member of the Bbsl complex, in an *I. auritulus* tick collected from a ground-foraging songbird in British Columbia (Table 6) [35]. Moreover, Banerjee et al. isolated *Borrelia bissettiae* (formerly *B. bissettii*; culture number 1340) from *I. angustus* larvae in southwestern B.C. [71]. Since *B. bissettiae* is cycling enzootically in this coastal area, it is likely that this borrelial species is dispersed by wild birds and attached ticks.

### 4.14. Epidemiological Significance of Ticks on Songbirds

It is noteworthy that three tick species (i.e., *I. affinis*, *I. dentatus*, and *I. minor*) were transported into central and eastern Canada during northward spring migration [14,59,60]; two of these extralimital ticks (*I. affinis* and *I. minor*) are known to be enzootic vectors of Bbsl. Although *I. affinis* and *I. minor* seldom bite humans, it appears that they are more important enzootic vectors of Bbsl in the southeastern U.S.A. than *I. scapularis* [36]. Of epidemiologic significance, Bbsl-infected, songbird-transported *I. scapularis* ticks have been documented as far west and as far north as Peace River, Alberta [72]. In the same study, Bbsl-infected *I. scapularis* nymphs were also reported in Atlantic Canada in Cape Breton Island, Nova Scotia, and the province of Newfoundland and Labrador. East of the Rocky Mountains, Bbsl-positive *I. scapularis* have been collected from songbirds in all provinces (Alberta, Saskatchewan, Manitoba, Ontario, Quebec, New Brunswick, Prince Edward Island, Nova Scotia, and Labrador and Newfoundland) [9,10,11,12,13,14,15,37,59,72]. These bird parasitisms underpin the fact that people do not have to visit an endemic area to contract Lyme disease.

We collected bird-rabbit ticks, *Haemaphysalis leporispalustris*, from songbirds and lagomorphs in several areas Canada-wide, and some of these ticks were positive for Bbsl. Our findings are consistent with Banerjee et al. who cultured Lyme disease spirochetes from *H. leporispalustris* collected in northwestern Alberta [73]. It is worth mentioning that *H. leporispalustris* larvae and nymphs are the predominant tick species that parasitize fall passerine migrants in central and eastern Canada. Of note, *H. leporispalustris*, a bird- and lagomorph-feeding ectoparasite, has transcontinental distribution in Canada [8]. Songbirds widely disperse *H. leporispalustris* immatures, and lagomorphs are present in each province to act as terrestrial hosts. Lagomorphs are competent reservoirs of Bbsl, especially for the genomospecies *B. andersonii* [74,75]. In Table 6, we have highlighted some of the significant *H. leporispalustris* parasitisms that were positive for Bbsl. Although a rare occurrence, *H. leporispalustris* is known to bite humans [76].

## 5. Conclusions

Our findings show that Bbsl has wide distribution across Canada, and multiple tick species are involved in the enzootic maintenance cycle of this highly adaptive spirochete. Of 18 species tested, 15 were Bbsl-positive by PCR. Our data provide many tick–host firsts for indigenous and extralimital ticks parasitizing wild birds and terrestrial mammals coast to coast. The groundbreaking discovery of *I. cookei* on Vancouver Island suggests that this tick species has undergone a major geographic shift in Canada. In addition, wild birds transport bird-feeding ticks to new foci during long-distance migrations. Of special note, a Bbsl-positive *A. longirostre* was transported from the Neotropics by a Willow Flycatcher. In all, 16 of the 27 tick species in this study are bird-feeding ticks, and the majority are known to harbor and transmit tick-borne pathogens. Notably, six tick species were positive for Bbsl within the southeastern region of Vancouver Island, and suggest that these ectoparasites are involved in a 6-tick, enzootic maintenance cycle of Bbsl. Not only do wild birds widely disperse Lyme disease vector ticks countrywide, terrestrial mammals maintain Bbsl in numerous localized Lyme disease foci. Notably, 6 of 15 tick species that were infected with Bbsl bite humans. Ultimately, healthcare professionals must be cognizant that multiple tick species and their vertebrate hosts perpetuate Lyme disease spirochetes and associated tick-borne pathogens. These tick-borne zoonoses have spawned a major public health crisis throughout Canada.

## Figures and Tables

**Table 1:** Tick species collected from avian and mammalian hosts in Canada, 2013–2016.

Tick Species	Ticks Collected	Bites Humans	Ticks Tested	Ticks Bbsl-pos.
*Amblyomma americanum*	5	Yes	NT	NT
*Amblyomma dissimile*	1	Occasional	NT	NT
*Amblyomma longirostre*	27	Rare	√	+ve
*Amblyomma maculatum*	3	Yes	NT	NT
*Amblyomma rotundatum*	1	Rare	NT	NT
*Dermacentor albipictus*	20	Rare	√	+ve
*Dermacentor andersoni*	16	Yes	NT	NT
*Dermacentor variabilis*	175	Yes	NT	NT
*Haemaphysalis leporispalustris*	178	Rare	√	+ve
*Ixodes affinis*	5	Rare	√	+ve
*Ixodes angustus*	37	Yes	√	+ve
*Ixodes auritulus*	88	No	√	+ve
*Ixodes banksi*	18	Rare	√	+ve
*Ixodes brunneus*	8	Rare	√	+ve
*Ixodes cookei*	65	Yes	√	+ve
*Ixodes dentatus*	2	Occasional	√	−ve
*Ixodes gregsoni*	17	No	√	+ve
*Ixodes marxi*	10	Yes	√	−ve
*Ixodes minor*	1	Rare	NT	NT
*Ixodes muris*	31	Yes	√	+ve
*Ixodes pacificus*	31	Yes	√	+ve
*Ixodes rugosus*	25	Rare	√	−ve
*Ixodes soricis*	1	Rare	NT	NT
*Ixodes spinipalpis*	43	Occasional	√	+ve
*Ixodes scapularis*	432	Yes	√	+ve
*Ixodes texanus*	18	Rare	√	+ve
*Ixodes uriae*	7	Yes	NT	NT
Total: 27	1265	14	18	15

NT, not tested; √, tick species tested; +ve, Bbsl positive; −ve, Bbsl negative.

**Table 2:** Detection of *Borrelia burgdorferi* sensu lato in *Ixodes* ticks collected from avian hosts in the Metchosin-Victoria-Vancouver-Maple Ridge, British Columbia area, 2013–2016.

Bird Species	Number of Ticks *Borrelia burgdorferi*-Positive Ticks/Number of Ticks Collected						
	No. of Hosts	*Ixodes auritulus*			*Ixodes pacificus*	*Ixodes spinipalpis*	Infection Rate (%)
		L	N	F	N	N	
American Robin, *Turdus migatorius* Linnaeus	7	0/4	1/6	7/13	0/0	0/0	8/23 (35)
Pacific Wren, *Troglodytes pacificus* Baird	3	0/1	1/2	0/0	0/0	1/1	2/4 (50)
Song Sparrow, *Melopiza melodia* (Wilson)	9	0/0	2/2	5/6	0/0	0/0	7/8 (88)
Common Yellowthroat, *Geothlypis trichas* (L.)	1	0/0	1/1	0/0	0/0	0/0	1/1 (100)
Fox Sparrow, *Passerella iliaca* (Merrem)	3	17/22	4/6	0/0	0/0	0/0	21/28 (75)
Hermit Thrush, *Catharus guttatus* (Palla)	2	0/5	0/0	0/0	0/0	0/0	0/5 (0)
Wilson’s Warbler, *Cardellina pusilla* (Wilson)	1	0/0	1/1	0/0	0/0	0/0	1/1 (100)
Varied Thrush, *Ixoreus naevius* Gmelin	1	0/0	1/1	0/0	0/0	0/0	1/1 (100)
Spotted Towhee, *Pipilo maculatus* Swainson	2	0/0	5/6	0/0	0/0	0/0	5/6 (83)
Steller’s Jay, *Cyanocitta stelleri* (Gmelin)	2	0/20	1/1	0/0	0/0	0/0	1/21 (5)
Purple Finch, *Haemorhous purpureus* (Gmelin)	1	0/0	0/0	0/0	0/0	0/1	0/1 (0)
Swainson’s Thrush, *Catharus ustulatus* (Nuttall)	3	0/0	0/0	3/3	0/0	0/0	3/3 (100)
House Wren, *Troglodytes aedon* (Vieillot)	1	0/0	0/0	0/0	0/1	0/0	0/1 (0)
California Quail, *Callipepla californica* (Shaw)	3	0/0	0/0	0/0	1/2	0/1	1/3 (33)
Cooper’s Hawk, *Accipiter cooperii* (Bonaparte)	1	0/1	0/0	0/1	0/0	0/0	0/2 (0)
Totals: 15	40	17/53	17/26	15/23	1/3	1/3	51/108 (47)

L, larva(e); N, nymph(s); F, female(s); Bb-pos., *Borrelia burgdorferi* sensu lato-positive.

**Table 3:** Detection of *Borrelia burgdorferi* sensu lato in *Ixodes* ticks collected from mammalian hosts in the Metchosin-Victoria-Vancouver-Maple Ridge, British Columbia area, 2013–2016.

Mammal Species	Number of *Borrelia burgdorferi*-Positive Ticks/Number of Ticks Collected																		
	No. of Hosts	*Ixodes angustus*				*Ixodes cookei*			*Ixodes pacificus*				*Ixodes spinipalpis*				*Ixodes texanus*		Infection Rate (%)
		L	N	M	F	L	N	F	L	N	M	F	L	N	M	F	N	F	
American red squirrel, *Tamiasciurus hudsonicus* (Erxleben)	4	0/0	0/2	1/3	3/18	0/0	0/0	0/0	0/0	0/0	0/0	0/0	0/0	0/0	0/0	0/0	0/0	0/0	4/23 (17)
American mink, *Neovison vison* (Schreber)	2	0/0	0/0	0/0	0/0	0/0	2/11	0/1	0/0	0/0	0/0	0/0	0/0	0/0	0/0	0/0	0/0	0/0	2/12 (17)
Brown rat, *Rattus norvegicus* (Berkenhout)	2	0/0	0/0	0/0	2/2	0/0	0/0	0/0	0/0	0/0	0/0	0/0	0/0	0/0	0/0	0/0	0/0	0/0	2/2 (100)
Columbian black-tailed deer, *Odocoileus hemionus columbianus* (Richardson)	2	0/0	0/0	0/0	0/0	0/0	0/0	0/0	0/0	0/0	0/1	0/3	0/0	0/0	0/0	0/0	0/0	0/0	0/4 (0)
Cottontail rabbit, *Sylvilagus floridanus* (J.A. Allen)	10	0/0	0/0	0/1	1/2	0/0	0/0	0/0	0/1	1/2	0/0	0/0	0/1	1/5	2/8	1/5	0/0	0/0	6/25 (24)
Deer mouse, *Peromyscus maniculatus* (Wagner)	1	0/0	0/0	0/0	0/0	0/0	0/0	0/0	0/0	0/0	0/0	0/0	0/3	2/2	0/0	0/0	0/0	0/0	2/5 (40)
Dog, domestic, *Canis lupus familiaris* (Linnaeus)	4	0/0	0/0	0/0	0/0	0/0	0/0	0/0	0/0	0/0	0/1	0/7	0/0	0/0	0/0	0/0	0/0	0/0	0/8 (0)
Douglas squirrel, *Tamiasciurus douglasii* (Bachman)	5	0/0	0/0	1/3	3/5	0/0	0/0	0/0	0/0	0/0	0/0	0/0	0/0	0/0	0/0	0/0	0/0	0/0	4/8 (50)
Human, *Homo sapiens* (Linnaeus)	6	0/0	0/0	0/0	0/0	0/0	0/0	0/0	0/0	0/1	0/0	0/4	0/0	0/1	0/0	0/0	0/0	0/0	0/6 (0)
Muskrat, *Ondatra zibethicus* (Linnaeus)	1	0/3	0/2	0/0	0/0	0/0	0/0	0/0	0/0	0/0	0/0	0/0	0/0	0/0	0/0	0/0	0/0	0/0	0/5 (0)
Pacific raccoon, *Procyon pacificus* (Merriam)	10	0/0	0/0	0/0	0/0	0/3	0/1	1/2	0/0	0/0	0/0	0/0	0/0	0/2	0/1	1/2	1/2	1/6	4/19 (21)
Snowshoe hare, *Lepus americanus* Erxleben	2	0/0	0/0	0/0	0/0	0/0	0/0	0/0	0/0	0/0	1/1	0/5	0/0	0/0	0/0	0/0	0/0	0/0	1/6 (17)
Striped Skunk, *Mephitis mephitis* (Schreber)	4	0/5	0/0	0/0	0/0	0/0	0/1	2/3	0/0	0/0	0/0	0/0	0/0	0/0	0/0	0/0	0/2	0/0	2/11 (18)
Western gray squirrel, *Sciurus griseus* (Ord)	5	0/0	0/0	0/0	2/4	0/0	0/0	0/0	0/0	0/0	1/1	1/1	0/1	0/0	0/0	1/2	0/0	0/0	5/9 (56)
Totals: 14	58	0/8	0/4	2/7	11/31	0/3	2/13	3/6	0/1	1/3	2/4	1/20	0/5	3/10	2/9	3/9	1/4	1/6	32/143 (22)

L, larva(e); N, nymph(s); M, male(s); F, female(s); Bb-pos., *Borrelia burgdorferi* sensu lato-positive.

**Table 4:** Detection of *Borrelia burgdorferi* sensu lato in *Ixodes* ticks collected from avian hosts in the London-St.Thomas-Simcoe-Toronto, Ontario area, 2013–2016.

Bird Species	Number of Ticks *Borrelia burgdorferi*-Positive Ticks/Number of Ticks Collected									
	No. of Hosts	*Ixodes affinis*	*Ixodes brunneus*		*Ixodes minor*	*Ixodes muris*		*Ixodes scapularis*		Infection Rate (%)
		N	N	F	L	L	F	L	N	
Hermit Thrush, *Cathrus guttatus* (Pallas)	4	0/0	0/0	1/1	0/0	0/1	0/0	0/0	3/3	4/5 (80)
Black-throated Blue Warbler, *Setophaga caerulescens* (Gmelin)	1	0/0	0/0	0/0	0/0	0/1	0/0	0/0	0/0	0/1 (0)
Blue Jay, *Cyanocitta cristata* (Linnaeus)	3	0/0	0/0	0/0	0/0	0/0	0/0	0/1	7/10	7/11 (64)
Common Yellowthroat, *Geothlypis trichas* (Linneaus)	5	0/0	0/0	0/0	NT/1	0/0	0/0	0/0	1/4	1/4 (25)
Swainson’s Thrush, *Catharus ustulatus* (Nuttall)	3	0/1	0/0	0/0	0/0	0/0	0/0	0/0	1/1	1/2 (50)
Tennessee Warbler, *Oreothlypis peregrina* (Wilson)	1	0/0	0/0	0/0	0/0	0/0	0/0	1/2	0/0	1/2 (50)
White-throated Sparrow, *Zonotrichia albicollis* (Gmelin)	3	0/0	0/1	0/0	0/0	0/0	0/1	0/0	0/1	0/3 (0)
Lincoln’s Sparrow, *Melospiza lincolnii* (Audubon)	1	0/0	0/0	0/0	0/0	0/0	0/0	0/0	0/1	0/1 (0)
Mourning Warbler, *Oporornis philadelphia* (Wilson)	1	0/0	0/0	0/0	0/0	0/0	0/0	0/0	1/1	1/1 (100)
Dark-eyed Junco, *Junco hyemalis hyemalis* (Linnaeus)	3	0/1	0/0	0/2	0/0	0/0	0/0	0/0	0/0	0/3 (0)
House Wren, *Troglodytes aedon* (Vieillot)	3	0/1	0/0	0/0	0/0	0/0	0/0	0/0	1/1	1/2 (50)
Baltimore Oriole, *Icterus galbula* (Linnaeus)	1	0/0	0/0	0/0	0/0	0/0	0/0	0/0	1/2	1/2 (50)
Gray-cheeked Thrush, *Catharus minimus* (Lafresnaye)	1	0/0	0/0	0/0	0/0	0/0	0/0	0/0	1/1	1/1 (100)
American Redstart, *Setophaga ruticilla* (Linnaeus)	1	0/0	0/0	0/0	0/0	0/0	0/0	0/0	0/1	0/1 (0)
Winter Wren, *Troglodytes hiemalis* Vieillot	1	0/0	0/0	0/0	0/0	0/1	0/0	0/0	0/0	0/1 (0)
Red-breasted Grosbeak, *Pheucticus ludovicianus* (Linnaeus)	1	0/0	0/0	0/0	0/0	0/0	0/0	0/0	0/1	0/1 (0)
Totals: 16	33	0/3	0/1	1/3	NT/1	0/3	0/1	1/3	16/27 (59)	18/41 (44)

L, larva(e); N, nymph(s); F, female(s); Bb-pos., *Borrelia burgdorferi* sensu lato-positive. NT, not tested.

**Table 5:** Detection of *Borrelia burgdorferi* sensu lato in *Ixodes* ticks collected from mammalian hosts in the London-St. Thomas-Simcoe-Toronto, Ontario area, 2013–2016.

Mammal Species	Number of *Borrelia burgdorferi*-Positive Ticks/Number of Ticks Collected													
	No. of Hosts	*Ixodes angustus*			*Ixodes cookei*			*Ixodes scapularis*				*Ixodes texanus*		Infection Rate (%)
		N	M	F	N	M	F	L	N	M	F	M	F	
Dog, domestic, *Canis lupus familiaris* Linnaeus	2	0/0	0/0	0/0	0/0	0/0	0/0	0/0	0/0	0/0	2/5	0/0	0/0	2/5 (40)
Cat, domestic, *Felis silvestris catus* Linnaeus	2	0/0	0/0	0/0	0/0	0/0	0/0	0/0	0/0	0/0	1/3	0/0	0/0	1/3 (33)
Striped skunk, *Mephitis mephitis* (Schreber)	4	0/0	0/0	0/0	1/2	0/0	1/3	0/0	0/0	0/0	0/0	0/0	0/0	2/5 (40)
Weasel, *Mustela erminea* Linnaeus	2	0/0	0/0	0/0	1/1	0/0	1/3	0/0	0/0	0/0	0/0	0/0	0/0	2/4 (50)
Eastern raccoon, *Procyon lotor lotor* Linnaeus	5	0/0	0/0	0/0	0/0	0/0	0/0	0/0	0/0	0/0	0/0	0/5	6/8	6/13 (46)
Human, *Homo sapiens* Linnaeus	4	0/0	0/0	0/0	0/0	0/0	0/0	0/0	0/0	0/2	1/2	0/0	0/0	1/4 (25)
American red fox, *Vulpes vulpes fulvus* Desmarest	3	0/0	0/0	0/0	0/3	0/0	1/2	0/0	0/0	0/1	2/3	0/0	0/0	3/9 (33)
American red squirrel, *Tamiasciurus hudsonicus* (Erxleben)	2	0/1	1/1	0/0	0/0	0/0	0/0	0/0	1/3	0/0	0/0	0/0	0/0	2/5 (40)
Groundhog, Marmota monax (Linnaeus)	2	0/0	0/0	0/0	1/2	0/0	1/3	0/0	0/0	0/0	0/0	0/0	0/0	2/5 (40)
Northern short-tailed shrew, *Blarina brevicauda* (Say)	1	0/0	0/0	0/0	0/0	0/0	0/0	2/3	1/2	0/0	0/0	0/0	0/0	3/5 (60)
Cottontail rabbit, *Sylvilagus floridanus* (J.A. Allen)	2	0/0	0/0	0/0	0/0	0/0	0/0	0/0	0/1	0/0	0/1	0/0	0/0	0/2 (0)
Eastern chipmunk, *Tamias striatus* (Linnaeus)	4	0/0	0/0	0/0	0/0	0/0	0/0	0/1	3/5	0/0	0/0	0/0	0/0	3/6 (50)
White-tailed deer, *Odocoileus virginianus* Zimmermann	1	0/0	0/0	0/0	0/0	0/0	0/0	0/0	0/0	0/1	1/3	0/0	0/0	1/4 (25)
Horse, *Equus caballus* Linnaeus	1	0/0	0/0	0/0	0/0	0/0	0/0	0/0	0/0	0/0	1/2	0/0	0/0	1/2 (50)
Muskrat, *Ondatra zibethicus* (Linnaeus)	1	0/2	0/0	0/0	0/0	0/0	0/0	0/0	0/0	0/0	0/0	0/0	0/0	0/2 (0)
Totals: 15	36	0/3	1/1	0/0	3/8	0/0	4/11	2/4	5/11	1/4	7/19	0/5	6/8	29/74 (39)

L, larva(e); N, nymph(s); M, male(s); F, female(s); Bb-pos, *Borrelia burgdorferi* sensu lato-positive.

**Table 6:** Tick–host records for birds and mammals in Canada, and *B. burgdorferi* sensu lato in ticks, by province, 2013–2016.

Geographical Location	Host Species	Tick Species	Life Stage	Collection Date	Epidemiological Significance
**Alberta (AB)**					
Calgary	Yellow-rumped Warbler	*I. spinipalpis*	N	30 May 2014	Bb-pos tick on a bird in AB
Pincher Creek	Snowshoe hare	*Hlp*	L, N	8 May 2014	Bb-pos tick in s. AB
Lethbridge	American Robin	*I. spinipalpis*	N	7 May 2015	HR in AB
**British Columbia (BC)**					
Metchosin	Cottontail rabbit	*I. spinipalpis*	M, F	7 February 2013	HR
North Saanich	Raccoon	*I. cookei*	L, N, F	26 February 2013	HR in BC
Burnaby	Striped skunk	*I. rugosus*	N	11 April 2013	HR
Colwood	Raccoon	*I. texanus*	F	13 June 2013	*I. texanus* on Vancouver Is
Beaver Lake	Mink	*I. cookei*	N, F	27 June 2013	Bb-pos *I. cookei* on mink in Canada
Burnaby	Striped skunk	*I. cookei*	F	5 July 2013	Bb-pos *I. cookei* on skunk in Canada
Gibsons	Douglas squirrel	*I. angustus*	F	1 September 2013	HR of *I. angustus* in Canada
Rocky Point	Pacific Wren	*I. auritulus*	N	31 August 2013	Bb-pos *I. auritulus* on this host
Abbotsford	Eastern gray squirrel	*I. angustus*	F	3 June 2013	HR
Burnaby	Eastern gray squirrel	*I. angustus*	F	18 September 2013	Bb-pos *I. angustus* on this host
Maple Ridge	Snowshoe hare	*I. pac.; I. spin.*	M, F; F	14 June 2013	HR, Bb-pos; HR, Bb-pos; co-infest
Metchosin	Pacific Wren	*I. auritulus*	L	22 June 2013	HR
Rocky Point	House Wren	*I. pacificus*	N	22 June 2013	HR
Ladysmith	California Quail	*I. spinipalpis*	N	25 June 2013	HR
Rocky Point	Vagrant shrew	*I. soricis*	F	3 September 2013	Southernmost collection in Canada
Kamloops	Bighorn Sheep	*D. andersoni*	M, F	20 May 2013	HR
East Sooke	Red squirrel	*I. angustus*	M, F	28 October 2013	Bb-pos *I. angustus* on red squirrel
Sechelt	Snowshoe hare	*I. pacificus*	M, F	26 March 2014	Bb-pos *I. pacificus* on this host
North Saanich	Cottontail rabbit	*I. spinipalpis*	M, F	5 May 2014	Bb-pos *I. spinipalpis* on this host
Kamloops	Moose	*D. andersoni*	M, F	25 April 2014	HR
Saanich	California Quail	*I. pacificus*	N	20 June 2014	Bb-pos *I. pacificus* on this host
Burnaby	Brown rat	*I. angustus*	F	13 September 2014	HR; Bb-pos *I. angustus* on this host
Weldon	Cat, domestic	*D. andersoni*	F	14 May 2014	HR
Rocky Point	Wilson’s Warbler	*I. auritulus*	N	1 August 2014	Bb-pos *I. auritulus* on this host
North Vancouver	Douglas squirrel	*I. angustus*	N, F	13 August 2014	Bb-pos *I. angustus* on this host
Coquitlam	Striped skunk	*I. angustus*	N	13 September 2014	HR
Burnaby	Spotted Towhee	*I. auritulus*	N	18 October 2014	HR; Bb-pos
Vancouver	Steller’s Jay	*I. auritulus*	N	23 October 2014	HR; Bb-pos
Merville	Cooper’s Hawk	*I. auritulus*	L, F	17 February 2015	*I. auritulus* on Cooper’s Hawk
Metchosin	Purple Finch	*I. spinipalpis*	N	24 April 2015	HR
Esquimalt	River otter	*I. rugosus*	N	29 April 2015	*I. rugosus* on Vancouver Is
Metchosin	Cottontail rabbit	*I. spinipalpis*	M, F	3 May 2015	*Borrelia lanei*, first in Canada [34]
Oak Bay	Raccoon	*I. spinipalpis*	F	3 August 2015	HR
Metchosin	Varied Thrush	*I. auritulus*	N	31 October 2015	*Borrelia americana*, first in Canada [35]
Saanich	Pacific Wren	*I. spinipalpis*	N	9 March 2015	HR
Victoria	Eastern gray squirrel	*I. spinipalpis*	L	26 March 2015	HR
Metchosin	Muskrat	*I. angustus*	L, N	9 April 2016	First report, tick on muskrat, Canada
Saanich	Cottontail rabbit	*I. ang*; *I. spin.*	F, N	12 April 2016	Co-infestation
Victoria	Cottontail rabbit	*I. ang*; *I. spin.*	L, L	27 May 2016	Co-infestation
Central Saanich	Cottontail rabbit	*I. spin.; I. ang*; *I. pac.*	M, F; N; N	6 September 2016	Triple co-infestation
Sooke	American mink	*I. cookei*	N	2 October 2016	HR, Vancouver Is.
**Manitoba (MB)**					
Morden	Snowshoe hare	*Hlp*	F	17 May 2014	HR in MB; Bb-pos
Gardenton	Red fox	*I. scapularis*	N, F	19 May 2014	HR in Canada
Gunton	Raccoon	*D. variabilis*	M, F	17 July 2014	HR in MB
Steinback	Groundhog	*I. cookei*	N	30 July 2014	HR in MB
**New Brunswick (NB)**					
Alma	Red fox	*I. scapularis*	N	23 June 2013	HR in NB; Bb-pos
Woodstock	Groundhog	*I. cookei*	N	9 July 2014	HR in NB
Fredericton	Southern red squirrel	*I. scapularis*	N	19 June 2016	HR in NB; Bb-pos
**Newfoundland and Labrador (NL)**					
Branch	Gray-cheeked Thrush	*Hlp*	N	27 July 2013	HR in NL
Gannet Island	Human	*I. uriae*	N, F	29 July 2013	HR
Corner Brook	Snowshoe hare	*Hlp*	F	20 May 2014	HR in NL
**Nova Scotia (NS)**					
Broad Cove	Raccoon	*I. scapularis*	N	17 June 2014	HR in NS; Bb-pos
Brighton	Southern red squirrel	*I. scapularis*	N	19 June 2014	HR; Bb-pos
St. Martins	Bovine	*I. scapularis*	N	23 June 2014	HR
Greenfield	Raccoon	*D. variabilis*	M, F	4 July 2014	HR in NS
Kemptville	Groundhog	*I. cookei*	N, F	22 July 2014	HR in NS
Lunenburg	Groundhog	*I. cookei*	N	28 July 2014	Bb-pos in NS
**Ontario (ON)**					
Long Point	Hermit Thrush	*I. brunneus*	F	18 April 2013	Bb-pos *I. brunneus*
Port Carling	Beaver	*I. banksi*	F	22 April 2013	Bb-pos *I. banksi*; in sc. ON
Oakville	Raccoon	*I. texanus*	F	5 May 2013	Bb-pos *I. texanus* in ON
Silver Lake	Beaver	*I. banksi*	N	15 May 2013	Bb-pos *I. banksi* in nw. ON
Toronto	Common Yellowthroat	*I. minor*	L	20 May 2013	HR, *I. minor* in Canada [36]
Toronto	Willow Flycatcher	*A. longirostre*	L	23 May 2013	Bb-pos *A. longirostre* in N. America
Long Point	Blue Jay	*I. scapularis*	N	15 May 2013	Bb-pos *I. scap.*, HR
Long Point	Swainson’s Thrush	*I. affinis*	N	24 May 2014	*I. affinis* in e. Canada
Belleville	Red fox	*I. cookei*	N, F	29 May 2013	*I. cookei* in e. Canada
Thunder Cape	Lincoln’s Sparrow	*I. den*; *I. scap.*	L; L, N	15 May 2013	Bb-pos *I. scap.* L & N; co-infest
London	Striped skunk	*I. cookei*	N, F	11 August 2013	Bb-pos *I. cookei* in eastern Canada
Ruthven Park	Tennessee Warbler	*I. scapularis*	L	1 September 2013	HR in N. America
Kenora	Snowshoe hare	*Hlp*	N	11 October 2013	Bb-pos *Hlp* in ON
Kenora	Dog	*D. albipictus*	N	9 December 2013	Bb-pos *D. albipictus*
Kenora	Human	*I. cookei*	N	10 May 2013	Bb-pos *I. cookei* on human, nw. ON
Kenora	Ermine	*I. gregsoni*	N, F	2 December 2013	HR; Bb-pos *I. gregsoni*
Long Point	Veery	*A. amer.*	L	22 May 2013	HR
Long Point	Magnolia Warbler	*A. long.*	L	24 May 2013	HR
Long Point	Acadian Flycatcher	*A. long.*	L	30 May 2013	HR
Thunder Cape	Chipping Sparrow	*I. scapularis*	N	23 May 2013	Bb-pos *I. scapularis* on this host
Long Point	Veery	*A. rotundatum*	N	26 May 2014	HR; first report on a bird [17]
Arthur	Human	*I. cookei*	N	25 April 2014	Bb-pos *I. cookei* on human
Barry’s Bay	Beaver	*I. banksi*	N, F	3 May 2014	Bb-pos *I. banksi* nymph, e. ON
Toronto	White-throated Sparrow	*I. brunneus*	N	1 May 2014	First record of *I. brunneus* nymph
Toronto	Common Yellowthroat	*I. den*; *I. scap.*	L; 2L, N	15 May 2014	Co-infestation
Toronto	House Wren	*I. scapularis*	N	15 May 2014	Bb-pos *I. scapularis* on this host
Toronto	Yellow Warbler	*A. long.*	L	18 May 2014	HR
Toronto	Gray-cheeked Thrush	*I. scapularis*	N	26 May 2014	Bb-pos *I. scapularis* on this host
Kenora	Red-backed vole	*I. muris*	F	1 June 2014	HR; Bb-pos
Kenora	Snowshoe hare	*Hlp*	N	3 June 2014	Bb-pos in ON
Long Point	Brown Thrasher	*I. scapularis*	N	1 June 2014	Bb-pos in Canada
St. Thomas	Short-tailed weasel	*I. cookei*	F	6 June 2014	HR; Bb-pos
Walsingham	Eastern chipmunk	*I. scapularis*	N	3 July 2014	Bb-pos
Keewatin	Dog, domestic	*I. muris*	F	7 July 2014	Bb-pos; HR with nw. ON
Fort Frances	Porcupine	*D. variabilis*	M, F	18 July 2014	HR in nw. ON
Kenora	Snowshoe hare	*Hlp*; *D.alb.*	L, N, F; N	28 July 2014	HR (*D. albipictus*); co-infest
Kenora	Red fox	*D. variabilis*	F	18 July 2014	HR in nw. ON
Keewatin	Northern short-tailed shrew	*I. muris*	F	23 July 2014	Bb-pos; HR in nw. ON
Kenora	Deer mouse	*I. angustus*	F	1 August 2014	Bb-pos; HR in ON
Verona	Human	*I. scapularis*	F	28 September 2014	Bb-pos in e. ON
Toronto	Hermit Thrush	*I. muris*	L	17 October 2014	HR; larval *I. muris*, s. ON
Kanata	Rusty Blackbird	*I. brunneus*	F	30 April 2015	HR; on this host
Ruthven Park	Chestnut-sided Warbler	*A. longirosre*	L	11 May 2015	HR; in N. America
Toronto	Swainson’s Thrush	*I. affin*; *I. scap.*	N, N	24 May 2015	Co-infestation; *I. scapularis* Bb-pos
Toronto	Veery	*A. dissimile*	N	24 May 2015	HR on bird in N. America [16]
Toronto	American Redstart	*I. scapularis*	N	26 May 2015	HR in Canada
Belmont	Eastern chipmunk	*I. scapularis*	N	9 June 2015	Bb-pos
Keewatin	Cat, domestic	*I. muris*	F	17 July 2015	Bb-pos; HR in nw. ON
Toronto	Black-throated blue Warbler	*I. muris*	L	28 August 2015	HR; larval *I. muris*, s. ON
Toronto	Winter Wren	*I. muris*	L	6 October 2015	HR; larval *I. muris*, s. ON
Toronto	Dark-eyed Junco	*I. muris*	F	20 October 2015	HR; larval *I. muris*, s. ON
Ruthven Park	Ruby-crowned Kinglet	*Hlp*	L	21 October 2015	HR; in Canada
Willard Lake	Marten	*I. marxi*	F	28 October 2015	HR; in Canada
Thunder Cape	Nashville Warbler	*I. scapularis*	N	27 October 2015	HR; in Canada
Anglican Island	Red fox	*I. cookei*	N	14 April 2016	HR; nw. ON
Long Point	Slate-colored Junco	*I. affinis*	N	17 April 2016	HR; in Canada
Ruthven Park	House Wren	*I. affinis*	N	28 April 2016	HR; in Canada
Long Point	Baltimore Oriole	*I. scapularis*	N	18 May 2016	HR; in Canada
Toronto	Red-breasted Grosbeak	*I. scapularis*	N	9 May 2016	HR; in Canada
Floradale	Dog, domestic	*A. mac.*	F	27 August 2016	HR; in Canada
Jim Lake	American mink	*I. gregsoni*	N	5 November 2016	Farthest west in Canada
Barry’s Bay	Fisher	*I. cookei*	F	31 December 2016	HR; in s. ON
**Prince Edward Island (PE)**					
Brudenell	Cat, domestic	*I. scapularis*	F	19 May 2014	HR in PE; Bb-pos
Montague	Eastern chipmunk	*I. scapularis*	N	19 June 2014	HR; in PE
Georgetown	White-throated Sparrow	*I. scapularis*	N	30 May 2015	HR; in PE; Bb-pos
**Québec (QC)**					
Ste-Anne-de-Bellevue	Common Grackle	*I. brunneus*	F	26 May 2014	HR; in Canada
Chicoutimi	Snowshoe hare	*Hlp*	N	17 June 2014	Bb-pos *Hlp* in QC
Ste-Anne-de-Bellevue	Canada Warbler	*Hlp*	L	24 August 2014	Bb-pos; reservoir host
Coaticook	Red fox	*I. scapularis*	N	17 June 2014	HR; in QC
Victoriaville	Groundhog	*I. cookei*	N	25 July 2014	Bb-pos; in QC
Ste-Anne-de-Bellevue	American Redstart	*I. muris*	L, N	14 August 2014	Bb-pos; in QC
Ste-Anne-de-Bellevue	House Wren	*I. muris*	F	31 August 2014	Bb-pos; in QC
Ste-Anne-de-Bellevue	Bay-breasted Warbler	*I. scapularis*	F	15 May 2015	HR; in N. America
Ste-Anne-de-Bellevue	Northern Waterthrush	*I. affin*; *I. scap.*	N, N	23 May 2015	Bb-pos *I. scapularis*; co-infestion
Ste-Anne-de-Bellevue	Common Yellowthroat	*I. affinis*	N	26 May 2015	Bb-pos; HR in QC
Ste-Anne-de-Bellevue	Veery	*I. scapularis*	N	26 May 2016	Bb-pos; in QC
St-Patrice-de-Sherrington	Brown Thrasher	*I. scapularis*	N	23 June 2016	HR; in QC
Mirabel	American Kestrel	*I. scapularis*	N	29 June 2016	HR; in Canada [37]
Keewatin	Meadow vole	*I. muris*	N	11 August 2016	HR; in nw. ON
Ste-Anne-de-Bellevue	Magnolia Warbler	*I. scapularis*	L	25 August 2016	HR; in Canada
**Saskatchewan (SK)**					
Oxbow	Snowshoe hare	*Hlp*	L, N	6 May 2014	HR; in SK
Saskatoon	Striped skunk	*D. variabilis*	F	1 July 2014	HR; in SK
Meadow Lake	Snowshoe hare	*Hlp*	F	19 May 2015	Bb-pos *Hlp* in SK

L, larvae; N, nymph(s); M, male(s); F, female(s). co-infest., co-infestation; e., eastern; sc., southcentral; s., southern; nw., northwest; HR, new host record; Bb-pos, *Borrelia burgdorferi* positive; *D. alb.*, *Dermacentor albipictus*; *Hlp*, *Haemaphysalis leporispalustris*; *I. affin*, *Ixodes affinis*; *I. ang*, *Ixodes angustus*; *I. den*, *Ixodes dentatus*; *I. pac.*, *Ixodes pacificus*; *I. scap*, *Ixodes scapularis*; *I. spin.*, *Ixodes spinipalpis*; *A. amer.*, *Amblyomma americanum*; *A. long.*, *Amblyomma longirostre*; *A. mac.*, *Amblyomma maculatum*.
